# Prevalence and predictors of inappropriate prescribing in outpatients with severe mental illness

**DOI:** 10.1177/20451253231211576

**Published:** 2023-11-22

**Authors:** Lisanne Koomen, Ilona van de Meent, Floor Elferink, Ingeborg Wilting, Wiepke Cahn

**Affiliations:** UMC Utrecht, Psychiatry, Heidelberglaan 100, Utrecht, 3584CX, The Netherlands; UMC Utrecht, Psychiatry, Utrecht, The Netherlands; UMC Utrecht, Psychiatry, Utrecht, The Netherlands; UMC Utrecht, Pharmacy, Utrecht, The Netherlands; UMC Utrecht, Psychiatry, Utrecht, The Netherlands

**Keywords:** medication review, potentially inappropriate prescribing, severe mental illness

## Abstract

**Background:**

Potentially inappropriate prescribing (PIP) is frequent in geriatrics and results in an increased risk for adverse effects, morbidity, mortality and reduced quality of life. Research on PIP in psychiatry has mainly focused on elderly patients and inpatients.

**Objectives:**

To determine the prevalence and the predictors of PIP of psychotropic medication in outpatients with severe mental illness.

**Design:**

This study is part of the Muva study, a pragmatic open Stepped Wedge Cluster Randomized Trial of a physical activity intervention for patients (age ⩾ 16 years) with severe mental illness.

**Methods:**

A structured medication interview, questionnaires on social functioning, quality of life and psychiatric symptoms, and BMI and waist circumference measurements were performed followed by a structured medication review. Patients were divided into groups: PIP *versus* no PIP. Between-group differences were calculated and a multivariate binary logistic regression was performed to examine predictors for PIP. A receiver operating characteristics analysis was performed to determine the area under the curve (AUC).

**Results:**

In 75 patients, an average of 5.2 medications of which 2.5 psychotropic medication was used. 35 (46.7%) patients were identified with PIP. Unindicated long-term benzodiazepine use was the most frequently occurring PIP (34.1%). Predictors of PIP were female gender [odds ratio (OR) = 4.88, confidence interval (CI) = 1.16–20.58, *p* = 0.03], number of medications (OR = 1.41, CI = 1.07–1.86, *p* = 0.02) and lower social functioning (OR = 1.42, CI = 1.01–2.00, *p* = 0.05). The AUC was 0.88 for the combined prediction model.

**Conclusion:**

The prevalence of PIP of psychotropic medication in outpatients with severe mental illness is high. It is therefore important to identify, and where possible, resolve PIP by frequently performing a medication review with specific attention to females, patients with a higher number of medications and patients with lower social functioning.

**Trial registration:**

This trial was registered in The Netherlands Trial Register (NTR) as NTR NL9163 on 20 December 2020 (https://trialsearch.who.int/Trial2.aspx?TrialID=NL9163).

## Graphical abstract

**Infographic:**
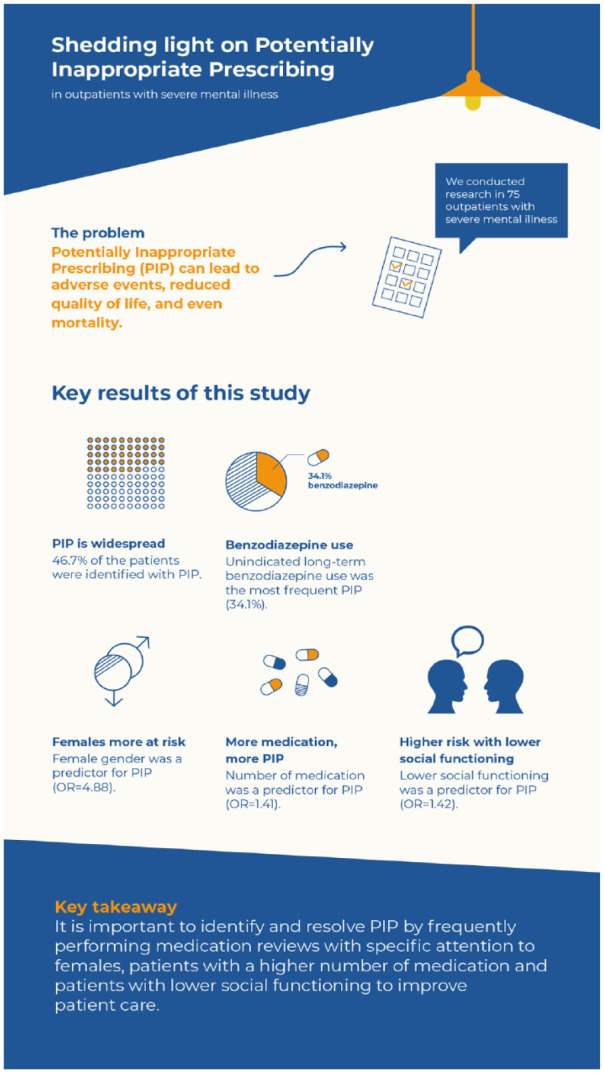

## Introduction

In the last decennium there is growing interest in appropriate prescribing and medication optimalisation.^1,2^ The focus of this research was primarily on geriatrics and shows that potentially inappropriate prescribing (PIP) occurs often and can result in increased prevalence of adverse effects, adverse drug events, morbidity, mortality, and reduced quality of life.^3,4^ Prescriptions are inappropriate if: they are misprescribed meaning that dosage, frequency or modality is inaccurate, overprescribed, meaning that there is no clinical indication, and underprescribed, meaning that potentially beneficial medication is missing.^5^

Research on PIP in psychiatry also mainly focused on elderly patients and showed PIP rates of 34–79% in elderly admitted patients.^6–11^ 91% in elderly outpatients,^12^ and 77.6% in elderly patients receiving 10 or more medications.^13^ The risk of PIP in elderly patients with mental disorders was associated with dementia,^7^ psychiatric comorbidity,^7^ lower daily functioning,^9^ length of hospital stay,^8^ polypharmacy,^12^ and number of prescribed drugs.^10,11^ Studies that investigated PIP in adult patients with mental disorders found PIP rates of 59% in adult inpatients,^14^ and a PIP rate of 56% in a nationwide cohort of antidepressant users in Denmark.^15^ The risk for PIP in adult patients with mental disorders was associated with somatic comorbidity,^14,15^ severity of depression,^16^ older age,^15^ being single,^15^ living in a rural area,^15^ polypharmacy,^17^ and number of prescribed drugs.^16,17^ Studies only focusing on PIP of psychotropic medication found a PIP rate of 55% in homeless people with schizophrenia and bipolar disorders^18^ and a PIP rate of 39% in chronic inpatients with mental disorders.^19^ PIP of psychotropic medication was associated with bipolar disorder,^18^ lower willingness to ask for help,^18^ and lack of lifetime history of psychiatric care.^18^ In most studies, PIP with a benzodiazepine or a Z-drug (zopiclone or zolpidem) was the most prevalent PIP.^8–12,20^

Above research shows high PIP rates and highlights the importance of appropriate prescribing and medication optimalization in patients with mental disorders. This is crucial since this population is already vulnerable. They have a 1.4–2 times increased risk of obesity, cardiovascular diseases, diabetes and a 15–20 years lower life expectancy and this is at least partly caused by psychotropic drug use.^21–23^ In addition, people with mental illness often experience long-lasting psychiatric symptoms and face difficulties in rehabilitation.^24,25^

The aim of this study was to determine the prevalence and predictors of PIP of psychotropic medication in adult outpatients with severe mental illness, receiving psychiatric ambulatory or primary care. To our knowledge, this has not been studied yet in this population. This population differs from an (chronic) inpatient setting. Appointments with practitioners are less frequent and medication reviews, especially in consultation with a pharmacist, are scarce. Yet, they often use psychotropics for extended periods. Therefore, the prevalence and predictors of PIP of psychotropic medication may differ from earlier studied psychiatric settings.

## Methods

### Study design and population

This study is part of the Muva study, a pragmatic open Stepped Wedge Cluster Randomized Trial of a physical activity intervention for patients with severe mental illness performed at a supported housing organization^26^ and the University Medical Centre Utrecht.^26^ This study used data from the baseline assessment, no data from the intervention period was used. Below the methods are shortly described according to the CONSORT reporting guidelines,^27^ and the full trial protocol of the Muva study is described elsewhere.^26^

A supported housing organization is an organization that supports patients with severe mental illness in their rehabilitation by giving assistance in their daily lives. The organization does not provide psychiatric treatment, all patients in this study received ambulatory care from a psychiatrist or received psychiatric care from their general practitioner. All 30 teams of the supported housing organization were invited to participate. The 12 teams that were willing to participate were divided into four clusters based on their location, and the order of the start of the intervention was randomized by drawing lots. The aim was to recruit 100 patients.

The mental health workers of the supported housing organization recruited patients that fulfilled the following inclusion criteria: age ⩾16 years, being diagnosed with a severe mental illness (i.e. a psychiatric disorder classified by the DSM-5 that causes limitations in psychosocial functioning for a duration of ⩾2 years), being able to give informed consent and to read and speak Dutch. Patients were not eligible for inclusion if they were unable to perform physical activity (defined as having a physical impairment that impedes physical activity). For this study we included participants from the Muva study that: used medication and gave permission for information retrieval from their general practitioner, psychiatrist and public pharmacy. This was because this information was needed to perform the structured medication review.

### Measurements

Patients were invited for the measurements in the University Medical Centre Utrecht. If they were unable to attend the University Medical Centre Utrecht, measurements were performed *via* videoconferencing. Sociodemographic information on gender and age was collected. A structured medication interview with the patient was performed on: medication use, side effects, if patients agreed with the medication regime and wishes regarding their current medication regimen.

Social functioning was measured with the Social Functioning Scale^28^ and five domains of social functioning were measured: social withdrawal, interpersonal communication, independency competence, independency performance, and recreation. A higher score indicates better social functioning. The WHO-QoL BREF^29^ was used to assess quality of life. The WHOQoL-BREF consists of 24 items divided into the domains physical health, psychological, social relationships, and environment and two general health items. The total score per domain is between 4 and 20, in which 20 indicates the highest score. The scores of the two general health items are between 1 and 5, with 5 indicating the highest score. The Brief Symptom Inventory^30^ was used to measure 53 psychiatric symptoms and the total number of symptoms present was calculated. Patients can score on a range of 0–53, with 0 meaning an absence of psychiatric symptoms and 53 meaning that all psychiatric symptoms are present. The Sint Hans Rating Scale^31^ was performed to evaluate movement disturbances. The Sint Hans Rating Scale is an evaluation tool for dyskinesia (score 0–8), parkinsonism (score 0–48), akathisia (score 0–12), and dystonia (score 0–6). It consists of 29 items divided into four dimensions of movement disturbances and a higher score indicates more complaints. In addition, length, weight and waist circumference were measured. Information on medical history, including psychiatric diagnoses, was retrieved from the general practitioner and/or psychiatrist. Polypharmacy was defined as using five or more different medications on Anatomical Therapeutic Chemical classification system (ATC) 3 level, excluding dermal preparations.^32^ Presence of psychiatric comorbidity was defined as having more than one psychiatric diagnosis.

### Medication review

Two pharmacists (a public pharmacist and a hospital pharmacist), a psychiatrist and a psychiatry resident reviewed the medication for the presence of PIP based on the clinical information that was retrieved. PIP of psychotropic medication was defined as psychotropic medication that is inappropriate if it was:

• Misprescribed meaning that dosage, frequency or modality was inaccurate:
  ○ Dosage too high or too low according to protocol recommendations, and the summary of product characteristics^33^
  ○ Frequency: too high or too low according to the summary of product characteristics^33^
  ○ Modality: for example, intravenous prescriptions for outpatients
  ○ Prescribed at the wrong time of the day (e.g. long-acting stimulant at the end of the day, or sedating antidepressant at the beginning of the day)

• Overprescribed, meaning that there was no clinical indication
• Underprescribed, meaning that potentially beneficial medication was missing

We used the Screening Tool for Older Person’s Prescriptions (STOPP) criteria, a screening tool for PIP in elderly, which is recommended by the Dutch geriatric and general physician guidelines^34^ and the Dutch psychiatric treatment guidelines (In The Netherlands there are treatment guidelines for groups of psychiatric disorders, e.g. psychotic spectrum disorders, depressive disorders, bipolar disorder.)^35^ to determine PIP of psychotropic medication. We used these guidelines, since there is no specific tool available for the screening of PIP of psychotropic medication. The results of the medication review were discussed with the physician (general practitioner or psychiatrist) of the patient, and adjusted when the physician gave new or additional information about patient’s medical history and status that required changes in the medication review.

If patients were unable to present themselves at the hospital for measurements, the medication review could still be performed if information on medication use and medical history was available.

### Outcomes

The primary outcome was the presence of PIP of psychotropic medication, and the secondary outcome was predictors of PIP.

### Statistical analysis

Variables were presented using measures of total number, percentages, means and standard deviation. Patients were divided into groups: PIP *versus* no PIP. Between-group differences were calculated using Analysis of Variance (ANOVA) for continuous variables and chi-square tests for categorical variables. Multivariate binary logistic regression using the Enter method was performed to examine predictors for PIP. Variables that were statistically significantly different in the between-group analyses, were included as independent variables in the model, with a maximum of six variables due to power constraints. For interpretation purposes the social functioning and quality of life outcomes were negatively decoded and the social functioning outcomes were measured in units of five. Results were presented using odds ratio and 95% confidence interval. After performing the multivariate binary logistic regression analysis, a receiver operating characteristics analysis was performed with the statistically significant predictors from the multivariate binary logistic regression analysis. This was done to determine the area under the curve (AUC) per predictor and for two combined models, to calculate the predictive value of the model. A *p* value of <0.05 was considered statistically significant. Analyses were performed using SPSS version 26.0.^36^

## Results

Table 1 displays the baseline characteristics and the results of the between-group analysis. A total of 75 patients were included. Data were not available for all clinical variables since six patients (8.0%) were not able to present themselves at the hospital for measurements and seven (9.3%) patients were not able to finish all the measurements. Mean age was 46.5 years old and 26 (34.7%) of the patients were female. A total of 22 patients (29.3%) were diagnosed with a psychotic disorder, followed by an autism spectrum disorder (21.3%), and 62.7% had psychiatric comorbidity.

**Table 1.** Baseline characteristics and results of analysis between groups.

Sociodemographic and clinical variables	Total *N* or mean (SD or %)	*N*	No PIP *N* or mean (SD or %)	*N*	PIP *N* or mean (SD or %)	*N*	*p* Value
Age (years)	46.45 (13.3)	75	44.28 (13.73)	40	48.94 (12.60)	35	0.13
Female	26 (34.7%)	75	9 (22.5%)	40	17 (48.6%)	35	**0.02**
Diagnosis				40		35	0.31
Psychotic disorder	22 (29.3%)	75	9 (22.5%)		13 (37.1%)		
Autism spectrum disorder	16 (21.3%)		11 (27.5%)		5 (14.3%)		
Personality disorder	10 (13.3%)		4 (10.0%)		6 (17.1%)		
Depressive disorder	7 (9.3%)		4 (10.0%)		3 (8.6%)		
Bipolar disorder	5 (6.7%)		2 (5.0%)		3 (8.6%)		
Substance abuse disorder	5 (6.7%)		5 (12.5%)		0 (0.0%)		
Posttraumatic stress disorder	4 (5.3%)		2 (5.0%)		2 (5.7%)		
Anxiety disorder	3 (4.0%)		1 (2.5%)		2 (5.7%)		
Attention deficit disorder	3 (4.0%)		2 (5.0%)		1 (2.9%)		
Known to have psychiatric comorbidity	47 (62.7%)	75	20 (50.0%)	40	27 (77.1%)	35	**0.02**
Treated by psychiatrist	49 (67.1%)	73	20 (51.3%)	39	29 (85.3%)	34	**<.01**
Number of medications	5.19 (13.33)	75	3.50 (2.38)	40	7.11 (3.76)	35	**<.01**
Number of psychotropic medications	2.47 (1.66)	75	1.35 (1.10)	40	3.74 (1.20)	35	**<.01**
Known to have polypharmacy	40 (53.33%)	75	13 (32.5%)	40	27 (77.1%)	35	**<.01**
Agreed with medication policy	61 (83.6%)	73	34 (87.2%)	39	27 (79.4%)	34	0.37
Experienced side effects	38 (54.3%)	70	15 (40.5%)	37	23 (69.7%)	33	**0.02**
Number of side effects	1.52 (1.99)	66	1.49 (1.99)	35	1.55 (2.01)	31	0.90
Believed medication is effective	54 (75.0%)	72	30 (78.9%)	38	24 (70.6%)	34	0.41
Had wishes regarding change of medication	35 (47.3%)	74	16 (41.0%)	39	19 (54.3%)	35	0.25
BMI in kg/m^2^	27.18 (6.91)	69	27.72 (5.29)	37	26.56 (8.45)	32	0.49
Waist circumference in cm	98.71 (18.60)	69	101.22 (15.94)	37	95.80 (21.20)	32	0.23
Sint Hans rating scale		70		38		32	
Dyskinesia	3.26 (5.68)		2.11 (3.51)		4.63 (7.32)		0.06
Parkinsonism	13.50 (7.67)		13.11 (6.92)		13.97 (8.58)		0.64
Akathisia	4.59 (3.18)		4.29 (2.60)		4.94 (3.18)		0.40
Dystonia	1.76 (1.97)		1.53 (1.66)		2.03 (2.29)		0.29
Social functioning scale		75		40		35	
Social withdrawal	97.38 (10.35)		98.69 (10.47)		95.89 (10.14)		0.25
Interpersonal communication	103.99 (9.79)		103.35 (9.18)		104.71 (10.53)		0.55
Independency competence	101.13 (10.36)		104.04 (7.03)		97.71 (12.52)		**0.01**
Independency performance	102.90 (9.43)		105.10 (8.45)		100.39 (9.99)		**0.03**
Recreation	113.89 (13.86)		117.90 (12.00)		109.30 (14.58)		**0.01**
Quality of life		70		40		31	
Quality of life	3.06 (1.03)		3.33 (0.97)		2.71 (1.01)		**0.01**
Experienced health	2.92 (1.23)		3.10 (1.24)		2.68 (1.19)		0.15
Physical health	12.63 (3.15)		13.43 (3.20)		11.60 (2.82)		**0.01**
Psychological health	12.32 (2.78)		13.12 (2.43)		11.32 (2.90)		**0.01**
Social relationships	12.98 (3.40)		13.04 (3.80)		12.90 (2.87)		0.87
Environment	14.30 (2.53)		15.10 (2.23)		13.28 (2.56)		**<.01**
Brief symptom inventory	26.03 (13.70)	75	23.80 (13.51)	40	28.57 (13.67)	35	0.13

BMI, body mass index; *N*, number; PIP, potentially inappropriate prescribing; SD, standard deviation.

On average, patients used 5.2 different medications and 2.5 psychotropic medications. A total of 35 patients (46.7%) were identified with one or more PIP of psychotropic medication. A total of 17 patients (20.7%) had two PIPs, one patient (1.2%) had three PIPs and two patients (2.4%) had four PIPs. Benzodiazepine use for longer than four consecutive weeks without an appropriate indication was the most frequent PIP (*n* = 28, 47.5%), followed by a combination of two benzodiazepines (*n* = 13, 22.0%) (Table 2). 38 patients (54.3%) experienced side effects and the most frequently mentioned side effects were fatigue (24.0%), weight gain (22.7%) and dry mouth (18.7%).

**Table 2.** Type and frequency of PIP.

PIP type	Frequency
Benzodiazepine use >4 weeks	28
More than 1 benzodiazepine	13
Benzodiazepine + sedating antihistaminic agent	7
Benzodiazepine + stimulant	4
Antipsychotic + antipsychotic	2
Benzodiazepine + low-dose quetiapine	2
Benzodiazepine + mirtazapine 7.5 mg	1
Sedating antihistamine + mirtazapine 7.5 mg	1
Sedating antihistamine + low-dose quetiapine	1
**Total**	59

PIP, potentially inappropriate prescribing.

Patients with PIP were more often female (48.6% *versus* 22.5%, *p* = 0.02), more often treated by a psychiatrist (85.3% *versus* 51.3%, *p* = <.01), suffered from psychiatric comorbidity (77.1% *versus* 50.0%, *p* = 0.02), used a higher number of medication (7.1 *versus* 3.5, *p* = <.01) and psychotropic medication (3.74 *versus* 1.35, *p* = <.01), had more polypharmacy (77.1% *versus* 32.5%, *p* = <.01). In addition, they experienced more side effects (69.7% *versus* 40.5%, *p* = 0.015), had lower social functioning on the scales’ independency competence (97.7 *versus* 104.0, *p* = 0.008), independency performance (100.4 *versus* 105.1, *p* = 0.03), and recreation (109.3 *versus* 117.9, *p* = 0.01) and had a lower quality of life (2.7 *versus* 3.3, *p* = 0.01) than patients without PIP.

Due to power constraints, we could not incorporate all statistically significant different variables from the between-group analyses into the multivariate binary logistic regression. Hence, we had to limit our selection to six independent variables. We prioritized the inclusion of the variable ‘number of medications’, as it provides a comprehensive measure encompassing both number of psychotropic medications and, when relevant, polypharmacy. In addition, with respect to social functioning we included the domain independence competency as this variable comprises the most clinical information, since this variable is about patient’s capability to function independently. For quality of life, the general health item ‘how would you rate your quality of life’ was included, since this is the most complete item of the WHO-QoL BREF. Moreover, the variables gender, psychiatric comorbidity, and physician type were included. Table 3 shows the results of the multivariate logistic regression analysis. Predictors of PIP were female gender, number of medications and a decrease in independency competency. Table 4 in the Supplemental Appendix shows the results of the multivariate binary logistic regression with the other domains of the social functioning scale.

**Table 3.** Results of the multivariate logistic regression.

Variables	Model PIP		
	OR	95% CI	*p* Value
Female *Reference is male*	4.88	1.16–20.58	**0.03**
Known to have psychiatric comorbidity *Reference is not known to have psychiatric comorbidity*	2.24	0.55–9.18	0.26
Treated by a general practitioner *Reference is treated by a psychiatrist*	0.31	0.08–1.28	0.11
Number of medications	1.41	1.07–1.86	**0.02**
Decrease in social functioning independence competency per 5 units	1.42	1.01–2.00	**0.05**
Decrease in quality of life	1.19	0.58–2.44	0.64
R^2^ Nagelkerke	0.52		

CI, confidence interval; OR, odds ratio for having PIP; PIP, potentially inappropriate prescribing.

**Figure 1.**
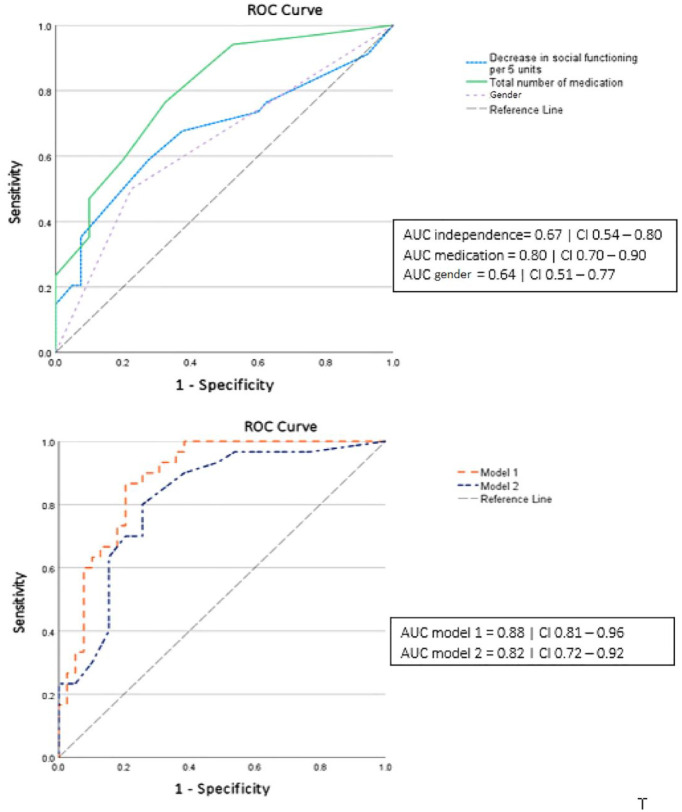
Results of the ROC analysis and the AUC. Model 1: gender, number of medications and decrease in social functioning. Model 2: gender and number of medications. AUC, area under the curve; ROC, receiver operating characteristics.

Figure 1 shows the results of the receiver operating characteristics analysis and the AUC of the independent variables and two combined models. It shows that the models ‘number of medications’, model 1 and model 2 are good in predicting PIP.

## Discussion

We identified PIP of psychotropic medication in 46.7% of the outpatients with severe mental illness, of which long-term benzodiazepine use was the most frequently identified PIP. Patients with PIP had more often psychiatric comorbidity, experienced more side effects, had lower social functioning and quality of life than patients without PIP. Predictors of PIP were female gender, number of medications and lower social functioning. The AUC showed that these variables combined were good predictors of PIP.

To our knowledge, this is the first study investigating the prevalence and the predictors of PIP of psychotropic medication in outpatients with severe mental illness. Previous studies on PIP of psychotropic medication found a PIP rate of 55% in homeless people with schizophrenia and bipolar disorders^18^ and a PIP rate of 39% in chronic inpatients with mental disorders.^19^ As hypothesized, the PIP rate in this study is higher (46.7%) than in the inpatient setting. This might be explained by the fact that outpatients have less appointments with their practitioner and medication reviews are scarce compared to an inpatient setting. Moreover, previous research in other psychiatric populations, both inpatient and outpatient settings, also found PIP with a benzodiazepine being the most prevalent PIP.^8–12,20^ This might be explained by several factors that hamper tapering of benzodiazepines. Benzodiazepines can cause physical dependency, and patients are strongly attached to benzodiazepines, which can create pressure for physicians to continue prescribing. Moreover, there is sometimes a lack of availability of other therapies, for example, insomnia cognitive behavioral therapy (In insomnia cognitive behavioral therapy the sustaining factors of sleeping problems, e.g. bad sleeping habits, physical stress and ruminating, are treated by behavioral interventions as sleep hygiene, sleep restriction, physical activity, and cognitive interventions as a rumination break and challenging thoughts.) or emotion regulation therapy, to treat the symptoms. In this situation, melatonin could be considered as an alternative for benzodiazepines. Additionally, patients and physicians can underestimate the adverse effects of long-term benzodiazepine use.^37^ In addition, in line with our results, studies investigating predictors of PIP of somatic and psychotropic medication in adult patients with mental disorders found the number of prescribed drugs to be predictors.^16,17^ Female gender was previously not found as a predictor for PIP in psychiatric patients. However, a systematic review^3^ on PIP in elderly patients without mental disorders found female gender to be a predictor of PIP. This finding is worrying since female patients^38^ face a higher risk of adverse effects since they have different pharmacokinetics and PIP increases the risk of adverse effects even more.^3,4^

Our study shows the importance of a frequent medication review in outpatients with severe mental illness. This is because high PIP rates of psychotropic medication were found, especially since patients with PIP experienced more side effects, and had lower social functioning and lower quality of life. In the medication review, extra attention should be paid to females, patients with a higher number of medications and patients with lower social functioning. Although the guidelines suggest yearly medication reviews, in clinical practice it is difficult to perform a medication review and practitioners experience several barriers. These barriers are a lack of time, lack of communication between the general practitioner, pharmacist and other involved practitioners, lack of a responsible practitioner who starts the process of a medication review, financial and capacity barriers and the absence of an electronic patient system linking all relevant data and that is accessible for all involved practitioners.^39^ We also experienced difficulties in performing the medication review. It was difficult to retrieve information on medical (psychiatric) history, and was time consuming to discuss the findings with all the practitioners. Most importantly, a specific guideline for deprescribing, such as the START/STOPP criteria,^34^ is missing for patients with mental disorders. Involvement and reimbursement of a clinical pharmacist in primary care and psychiatric outpatient teams might facilitate frequent medication reviews, decrease PIP and improve patient outcomes.^40^ Evidence from Slovenia shows that it is feasible and cost-effective.^41^

The lack of a medication review might not be the only reason why PIP rates of psychotropic medication, and unindicated long-term benzodiazepine use, are high. PIP in psychiatry differs from PIP in geriatrics, since PIP, with respect to continuation without indication, also occurs due to fear of relapse in the minds of patients, caregivers as well as prescribers. Patients with severe mental illness often have a long course of disease history with relapses and their medication regime is often the result of several attempts to stabilize their symptoms. Practitioners, caregivers and patients are therefore sometimes reluctant to change and deprescribe medication.^19,37,42,43^ The fear for a relapse is not unfounded,^44^ and for some patients it may be better to continue PIP as it may improve their wellbeing. Thus, in order to reduce high PIP rates and long-term benzodiazepine use, not only frequent medication reviews might be needed, but also improved availability of other therapies to treat symptoms, a better understanding of for which patients deprescribing can be successful, and a guideline for deprescribing of psychotropic drugs.

### Strengths and limitations

The strength of this study is that we not only reviewed patients’ charts, but also assessed mental and physical measures to inform the multidisciplinary medication review. Limitations are that we did not have full access to the medical records, therefore full information on somatic comorbidity was not available and we were unable to review the somatic medication on PIP. PIP in people with severe mental illness might be linked to their somatic comorbidities; thus, we might have missed cases of underprescription and overprescription of drugs for somatic illnesses. Moreover, despite having adequate information on the mental health condition for conducting medication reviews, potential cases of underprescription of psychotropic drugs may have been overlooked due to the unavailability of complete medical records and information about disease progression. Additionally, clinical diagnoses were retrieved from the medical records, and no instrument was used to assess patient’s diagnosis. Because we did not have full access to clinical information, we discussed the results of the medication review with the patient’s physician to optimize the review. Another limitation is that we examined medication prescription lists, and not medication administration lists that are used in inpatient settings. Therefore, we did not have information on how often benzodiazepines that were prescribed as ‘on demand’ were actually taken by the patient; thus patient’s actual medication use might differ. Moreover, the sample size of this study is limited, therefore we were unable to include all variables that differed between groups in the multivariate binary logistic regression. We were able to estimate a good prediction model for PIP, but future studies with larger sample size should be performed to confirm and validate this model.

In conclusion, PIP of psychotropic medication was found in 46.7% of the outpatients with severe mental illness. Patients with PIP had more often psychiatric comorbidity, experienced more side effects and had lower social functioning and quality of life. Predictors of PIP were female gender, number of medications and lower social functioning. We advise to perform a yearly medication review to identify PIP and where possible, resolve PIP in order to optimize care and rehabilitation. Extra attention should be paid to females, patients with a higher number of medications and patients with lower social functioning.

## Supplemental Material

sj-docx-1-tpp-10.1177_20451253231211576 – Supplemental material for Prevalence and predictors of inappropriate prescribing in outpatients with severe mental illness Click here for additional data file. Supplemental material, sj-docx-1-tpp-10.1177_20451253231211576 for Prevalence and predictors of inappropriate prescribing in outpatients with severe mental illness by Lisanne Koomen, Ilona van de Meent, Floor Elferink, Ingeborg Wilting and Wiepke Cahn in Therapeutic Advances in Psychopharmacology

sj-docx-2-tpp-10.1177_20451253231211576 – Supplemental material for Prevalence and predictors of inappropriate prescribing in outpatients with severe mental illness Click here for additional data file. Supplemental material, sj-docx-2-tpp-10.1177_20451253231211576 for Prevalence and predictors of inappropriate prescribing in outpatients with severe mental illness by Lisanne Koomen, Ilona van de Meent, Floor Elferink, Ingeborg Wilting and Wiepke Cahn in Therapeutic Advances in Psychopharmacology

## Declarations

**Ethics approval and consent to participate:** This study was approved by the Medical Ethical Committee of the University Medical Centre Utrecht (20-628/C). All patients were asked for verbal and written consent to participate.

**Consent for publication:** Not applicable.

**Availability of data and materials:** Data is available upon reasonable request.
