# Supplementary material for: Prevalence and predictors of inappropriate prescribing in outpatients with severe mental illness
Source: Ther Adv Psychopharmacol. 2023 Nov 22;13:20451253231211576. doi: 10.1177/20451253231211576 (PMC10666674; doi:10.1177/20451253231211576)
Supplement: sj-docx-1-tpp-10.1177_20451253231211576 – Supplemental material for Prevalence and predictors of inappropriate prescribing in outpatients with severe mental illness [file sj-docx-1-tpp-10.1177_20451253231211576.docx]

**Appendix**

|  | **Model with social functioning variable independence performance** | | | **Model with social functioning variable recreation** | | |
| --- | --- | --- | --- | --- | --- | --- |
|  | **OR** | **95% CI** | **p-value** | **OR** | **95% CI** | **p-value** |
| Female | 5.63 | 1.27 – 24.93 | **0.023** | 4.46 | 1.10 – 18.13 | **0.037** |
| Known with psychiatric comorbidity | 2.82 | 0.64 – 12.47 | 0.171 | 1.85 | 0.46 – 7.49 | 0.387 |
| Treated by a general practitioner | 0.02 | 0.04 – 0.88 | **0.034** | 0.40 | 0.10 – 1.51 | 0.176 |
| Number of medication | 1.28 | 0.97 – 1.69 | 0.081 | 1.36 | 1.04 – 1.78 | **0.025** |
| Decrease in social functioning independence performance per 5 units | 1.67 | 1.09 – 2.58 | **0.020** | - | - | - |
| Decrease in social functioning recreation per 5 units | - | - | **-** | 1.21 | 0.94 – 1.56 | 0.140 |
| Decrease in quality of life | 1.45 | 1.09 – 2.58 | 0.302 | 1.48 | 0.75 – 2.94 | 0.258 |
| R^2^ Nagelkerke | 0.543 | | | 0.496 | | |
| **Table 4. Results of the** **multivariate logistic regression.** CI = confidence interval, OR = odds ratio, PIP = potentially inappropriate prescribing | | | | | | |
