# Supplementary material for: Prevalence and predictors of inappropriate prescribing in outpatients with severe mental illness
Source: Ther Adv Psychopharmacol. 2023 Nov 22;13:20451253231211576. doi: 10.1177/20451253231211576 (PMC10666674; doi:10.1177/20451253231211576)
Supplement: sj-docx-2-tpp-10.1177_20451253231211576 – Supplemental material for Prevalence and predictors of inappropriate prescribing in outpatients with severe mental illness [file sj-docx-2-tpp-10.1177_20451253231211576.docx]

**CONSORT flow diagram**

**Dropout:**

- Did not use medication (n=3)
- Did not give consent for information retrieval (n=2)

**Dropout:**

- Did not use medication (n=4)
- Did not give consent for information retrieval (n=2)
- No time (n=2)
- No interest (n=2)
- Takes too much energy (n=2)

Control group
(n=10)

Intervention group
(n=65)

This paper

Control group
(n=15)

Intervention group
(n=77)

Allocation to the intervention
(n=101)

Assessed for eligibility (n=101)

Randomized
(n=101)

Baseline

Allocation

Enrollment
